# Nutrition Screening, Reported Dietary Intake, Hospital Foods, and Malnutrition in Critical Care Patients in Malawi

**DOI:** 10.3390/nu13041170

**Published:** 2021-04-01

**Authors:** Grace C. Barcus, Peggy C. Papathakis, Andrew Schaffner, Bernadette Chimera

**Affiliations:** 1 Department of Food Science and Nutrition, California Polytechnic State University, San Luis Obispo, CA 93407, USA; Gbarcus@calpoly.edu; 2 Queen Elizabeth Central Hospital, P.O. Box 95, Blantyre, Malawi; Bchimera@medcol.mw; 3 Department of Statistics, California Polytechnic State University, San Luis Obispo, CA 93407, USA; Aschaffn@calpoly.edu; 4 School of Public Health and Family Medicine, College of Medicine, University of Malawi, Chichiri, Blantyre, Malawi; 5 Kamuzu Central Hospital, Area 33 Mzimba Street, P.O. Box 106, Lilongwe, Malawi

**Keywords:** hospital malnutrition, reported reduced dietary intake, nutrition screening, MUAC

## Abstract

In low-income countries there are few data on hospital malnutrition. Reduced food intake combined with nutrient-poor foods served in hospitals contribute to nutritional risk. This study investigated whether reported dietary intake and disease state of hospitalized adults in critical care units was related to malnutrition determined by mid-upper arm circumference (MUAC). Adult in-patients (*n* = 126) in tuberculosis, burn, oncology, and intensive care units in two public tertiary hospitals in Malawi were screened for nutritional status using MUAC and a question on current dietary intake. The hospital menu was reviewed; portion sizes were weighed. The prevalence of moderate and severe malnutrition was 62%. Patients with organ-related diseases and infectious diseases had the highest rates of reduced reported dietary intake, 71.4% and 57.9%, respectively; however, there was no association between reported dietary intake and MUAC. In those unable to eat, however, the rate of severe malnutrition was 50%. The menu consisted of porridge and thickened corn-based starch with fried cabbage; protein foods were provided twice weekly. There was a nutrient gap of 250 calories and 13 gm protein daily. The findings support the need for increasing dietetic/nutrition services to prevent and treat malnutrition in hospitals using simple screening tools.

## 1. Introduction

In Africa, 25 to 72.3% of hospitalized patients are at risk for malnutrition [1,2,3]. Hospital malnutrition is a concern globally, however there are limited studies on the prevalence of hospital-based malnutrition in low resource countries, like Malawi. Hospital-based malnutrition is related to decreased food/fluid intake, altered nutrient metabolism due to severe injury and disease-associated inflammatory conditions, and nutrient losses from malabsorption or vomiting [4]. These conditions are commonly seen in critical care patients and are associated with poor nutritional status and therefore, poor health outcomes which include reduced immune function, impaired wound healing, muscle wasting, prolonged hospital stays, high treatment costs, and increased mortality [5,6,7,8]. Although the consequences of hospital malnutrition are severe, hospital-based malnutrition is still regularly underdiagnosed and untreated [9]. In order to prevent this, patients at risk for malnutrition need to be identified early in their hospital stay to ensure prompt and adequate nutritional support [10].

Non-mandatory nutrition screening, limited availability of nutrition professionals, and reduced dietary intake are associated with an increased risk for hospital malnutrition [11]. Requisite nutritional screening at admission is a simple approach to quickly identify patients at risk for malnutrition [11]. Nutrition screening is common in high resource settings, but is not mandatory in Malawi. Nutritional screening conducted by other health professionals in Malawi and other similar countries would allow for prompt referral to Registered Dietitians to focus on nutritional interventions for those at highest risk. Hospitalized patients, especially those who are critically ill and metabolically stressed, have increased requirements for nutrient rich foods to aid in the prevention and management of malnutrition [12,13]. Reported reduced dietary intake, commonly part of nutrition screening tools [14,15], can be related to poor quality hospital meal service, loss of appetite, change in protein and energy homeostasis due to disease state, inability to tolerate an oral diet, or lack of alternative feeding options [16,17]. Globally, mid-upper arm circumference (MUAC) is commonly used in children attending community malnutrition clinics [18,19,20,21], during pregnancy [22,23,24,25], and has been used to assess nutritional status in hospitalized patients [26,27]. In Malawi, however, MUAC is not routinely used in hospitalized adults outside of study settings, although there are no internationally recognized cut-off points for MUAC to define undernutrition in adults [28]. In Malawi, patient meals are provided through hospital catering services, which has limited capacity for diverse diets, which makes it difficult to meet the nutritional needs of patients for optimal recovery. Clinical dietetic professionals are increasing in Malawi due to new training programs and there is a critical opportunity for the prevention and treatment of hospital malnutrition, however, barriers such as a lack of nutrition screening and inadequate hospital meal service still stand in the way of achieving optimal nutrition in hospitalized patients.

The aim of this secondary analysis was to determine whether reported change in dietary intake combined with disease and MUAC measurement could be a useful and easy tool for non-nutrition health care providers to accurately characterize hospitalized patients for malnutrition risk. In addition, this analysis sought to determine the relationship between disease categories and change in dietary intake and malnutrition, and whether the macronutrient content of the meals provided by hospital catering services were adequate for patients in critical care units.

## 2. Materials and Methods

### 2.1. Study Design

This secondary analysis is from a larger hospital audit of nutrition status and management of critical care patients in Malawi, whose primary aim was to evaluate the rate of hospital malnutrition and how patients with high nutritional needs (critically ill patients) are managed in the hospital. It included the nutritional assessment and screening of patients, identified the types and amounts of food served in the hospital, and whether the hospitals or units have clear steps on how to identify and provide nutrition care to critically ill patients. This secondary analysis focused on the relationship between reported dietary intake and the prevalence of malnutrition determined by MUAC and further assessed by medical diagnosis. In addition, the nutritional quality and quantity of the food provided by hospital catering from the primary audit was assessed.

### 2.2. Study Participants

The survey was conducted from October to November 2019 in two public tertiary hospitals, Queen Elizabeth Central Hospital (QECH) and Kamuzu Central Hospital (KCH), in Malawi, Africa. Both adults and children were observed in the primary study, but for the purpose of this secondary analysis, only the results from the adults are reported. The sample size consisted of 126 male and female inpatients from the adult tuberculosis (TB), burn, oncology, high dependency units (HDU), and intensive care units (ICU). All male and female patients, 18 years or older, present in these units were eligible for this secondary analysis. To be selected for the primary study, participants needed to be present in the identified units on the days of assessment; the only exclusion criteria were if the patient or caregiver refused participation in the survey. The distribution of participants amongst hospitals and units was based on hospital capacity and average monthly patients seen per unit. The sample size was based on the Cochran formula, using a malnutrition prevalence of 25%, for a sample size of 108. To account for loss of information due to any reason, the sample size was adjusted for a loss to follow up rate of 15% (*n* = 124). Out of the 126 adult participants recruited for the study, the final sample size was 100 adult participants, due to missing data collection forms on 20 patients and six patients with incomplete Subjective Global Assessment (SGA) forms. For descriptive analysis, the sample size was 100 adult participants, however for modelling (*n* = 91) eight patients without MUAC and one without notation of sex were excluded. Of adult participants, 35% were from KCH and 65% were from QECH. The study protocol was reviewed and approved by the University of Malawi, College of Medicine Research and Ethics committee, and from the Institutional Review Board at California Polytechnic State University, San Luis Obispo. All participants and their guardians were provided written and verbal informed consent in Chichewa or English depending on the participant preference. For participants who could not sign their name, a thumbprint was used.

### 2.3. Demographic, Anthropometric, and Diet History Assessment

Participants’ demographic and medical history information, including age, gender, and medical diagnosis were collected from the medical record. Anthropometric measurements of weight, height, when available, BMI, and MUAC were obtained by trained nutrition staff members. Due to lack of equipment or inability for patients to stand, height and therefore, BMI were not measured in patients at QECH. The SGA, which included questions on weight, appetite, tolerance to food and fluids, dietary intake, and a nutrition-focused physical exam were completed for every participant. For the purpose of this paper, we will focus only on the response to dietary intake questions of the SGA. SGA is a validated clinical technique that assesses nutritional status based on an assessment that includes history of recent intake, weight change, gastrointestinal symptoms, and a clinical evaluation [29,30].

Change in dietary intake was assessed through one question on the SGA form, “Change in intake (duration and degree); base rating on current and prior status.” The answer choices were “(a) unable to eat, starvation,” “(b) borderline, decreasing,” or “(c) intake good, or no change, or slight change, short duration.”

For the MUAC, two independent measurements on the left side were taken and if the difference between the two measurements was more than 2 mm, a third measurement was obtained. The mean measurement was used in the analysis. If three measurements were taken, the mean of the closest pair was used. Malnutrition was classified based on MUAC measurement using the criteria: <23 cm, severely malnourished; 23–25.4 cm, moderately malnourished; ≥25.5 cm, adequately nourished [28,31,32,33,34,35].

The primary medical diagnosis per patient was used for categorization into one of four different disease categories: Burns + Surgical, Cancer, Infectious Disease, and Organ-Related Disease.

### 2.4. Nutrition Screening Protocols, Catering Services, and Nutritional Analysis of Foods Served

In each medical unit where high nutritional risk patients were surveyed, the presence of and compliance to nutrition screening and clinical guidelines was determined by asking the head nurse and recording the written or general guidance. To determine the foods offered and to characterize the quality of the hospital food services menu and the foods prepared and served, the hospital menu was obtained from the head of Catering Services for the previous eight weeks. In addition, portion sizes were weighed prior to being served and recipes were obtained from the cooks. The energy and macronutrient content of meals were analyzed using the Malawian Food Composition Table [36]. Nutrition study staff observed the daily meal services to weigh portion sizes of meals served. Additionally, due to the variation in hospital donations, the protein source varied, which means the nutritional content of the protein source does as well. For this reason, non-protein days were used for a representative analysis. Daily energy and macronutrients served were estimated and compared against estimated energy and diet diversity requirements for Malawian adults recommended by the World Food Program [37]. For protein, 1.2 gm/kg body weight was used [38].

### 2.5. Statistical Analysis

Data was entered into Microsoft Excel (2016) and analyzed using JMP Pro 15 (JMP^®^, Version *15*. SAS Institute Inc., Cary, NC, USA, 1989–2019). The data entered into excel were verified by a second team member. Data was screened for outliers. All available data were used in the analyses. Quantitative response data were summarized using means and standard deviation and analyzed using independent-sample t-tests and general linear models with normal errors. Qualitative response data were summarized using percentages and Chi-squared or Fisher Exact tests were used in analyses. For understanding the relationship of MUAC with explanatory variables hospital, sex, age, disease category, and dietary intake, logistic and linear models were fit that included main effects for all predictors as well as an interaction term the predictors disease category and dietary intake. Normality for quantitative responses in models was assessed using the Shapiro–Wilk test. Statistical significance criteria was set α = 0.05 and confidence level of 95% for all tests and intervals.

## 3. Results

### 3.1. Demographic, Anthropometric, and Medical Diagnosis

Of the 100 adult participants, the mean age was 40.6 ± 14.6 years, with most in the 35–50 year category; almost 60% of participants were male. Determined by MUAC, the prevalence of mild to no malnutrition was 38%, moderate malnutrition was 20.7%, and severe malnutrition was 41.3%, which was similar for both hospital locations, however only 11% had a malnutrition diagnosis documented in their medical records. The anthropometrics and demographics of participants are shown in Table 1. There were 44 unique medical diagnoses in this sample population and these were collapsed into Burns + Surgical (32%), Cancer (27%), Organ-Related Disease (21%), and Infectious Disease (20%). Table 2 shows the individual medical diagnoses in each disease category, along with sex and age by disease category.

### 3.2. Diet History Assessment

Over half of the patients, 57.6%, reported reduced dietary intake. Of those, 81.1% had borderline or decreasing dietary intake and 18.9% were reported being unable to eat. The MUAC measurements were consistent with a normal distribution (Shaprio-Wilk’s *p* = 0.59); mean MUAC measurement was 23.89 cm as shown in Table 1. Nearly 62% had moderate to severe malnutrition.

The rates of moderate to severe malnutrition were as follows, across the disease categories: Infectious Disease (68.4%), Organ-Related Disease (61.9%), Burns + Surgical (60%), and Cancer (59.2%). Mean MUAC measurements for hospital (QECH vs. KCH), sex, reported dietary intake, and disease categories are found in Table 3. Although not statistically significantly different, the mean MUAC measurement was lowest for Infectious Disease, followed by Cancer, Burns + Surgical, and Organ-Related Disease. Surprisingly, only 5.43% of patients who were severely malnourished were unable to eat while patients with a reported dietary intake of “borderline, decreasing” had the highest rate of severe malnutrition. The majority of participants with no change in dietary intake, however, were adequately nourished.

Using a single test to avoid inflated Type I error and maximize statistical power, there was no statistically significant relationship between MUAC and any of the hospitals (QECH vs. KCH), age, sex, medical diagnosis, reported dietary intake, and the interaction between medical diagnosis and reported dietary intake (F (14,76) = 0.54, *p*-value = 0.90); shown in Table 4.

In every disease category, over half of the patients had decreased reported dietary intake (Table 5). Patients with Organ-Related Diseases and infectious diseases had the highest rates of reduced reported dietary intake, 71.4% and 57.9%, respectively. Of all participants that reported reduced dietary intake, the disease categories Burns + Surgical and Infectious Disease had the highest rates of moderate to severe malnutrition: 84.65% and 72.7%, respectively.

Additionally, using MUAC group as a response in a nominal logistic regression, there was no statistically significant relationship between MUAC group and any of the hospitals (QECH vs. KCH), age, sex, medical diagnosis, reported dietary intake, and the interaction between medical diagnosis and reported dietary intake (overall model *p*-value = 0.667); shown in Table 6. There was, however, a positive trend for reported poor dietary intake in those with a severely malnourished MUAC score. This trend was shown by contingency analysis, where each reported dietary intake score was compared to the proportion of individuals with that score who were severely malnourished. With a reported dietary intake of no change, the proportion of individuals with a severely malnourished MUAC score was 33%. With borderline or decreasing reported dietary intake, the proportion of individuals with a severely malnourished MUAC score increased to 46.51%. Finally, with a dietary intake of “unable to eat”, the proportion of individuals with a severely malnourished MUAC score increased to 50%.

### 3.3. Nutrition Screening Protocols, Catering Services, and Nutritional Analysis of Foods at QECH

There were no formal, informal or written nutrition screening or intervention protocols on any TB, burns, oncology, surgical, HDU or ICU ward. In the TB, burn and oncology units, however, high protein porridge was offered twice a day to all patients. Additionally, two catering service staff members, with little nutritional training, screened for patients at nutritional risk twice a week. To be considered “at risk”, patients must present with burns, large open wounds, fractures, appear “wasted”, or be referred from other hospital staff. Depending on the unit, these patients would receive either a high protein diet or an extra high protein diet. 

Every patient was served three meals a day by the catering department. The menu consisted of porridge for breakfast, and twice a day, nsima, a thickened corn-based starch with fried cabbage. The only variation over the course of 2 months was the source of protein (soya pieces, legumes, egg, or beef) provided two times a week, depending on availability; fruit was never served. There are three diets available, normal, high protein, and extra high protein. The high protein diet consisted of the patient receiving one to two servings of whole milk in 250 mL sachets. Patients on the extra high protein diet received high protein porridge, milk and an additional portion of eggs, beans or meat when available. One of the challenges with these diets is that supplemental beef, milk, and eggs were mainly donated foods and available intermittently. If able, families of patients were advised to supplement the hospital diet with food from home, such as eggs, milk, and other fruit and vegetables.

The average nutritional composition of the hospital menu and individual diets are listed below in Table 7 and Table 8. The menu provided by the hospital does not provide any fruits or vegetables other than cabbage, and is low in dairy, whole grains, and bioavailable protein. Macronutrient requirements for adults vary by age, disease-state, gender, weight, and other different variables. Malawi guidance suggests a usual dietary intake for adults of 2205 kcal/day for adults [37]. Assuming that the patient consumes all food provided, the normal hospital diet provides 88.4% of estimated energy needs, representing a gap of 250 calorie per day. Using a conservative goal of 1.2 gm protein per day, the normal diet provides 80% of estimated protein requirement or a gap of 13 gm protein daily. The high protein diets have a gap of about 100 calories/day and 3–9 gm/day gap in protein intake.

## 4. Discussion

Nutritional risk screening is recommended and should be performed in patients at hospital admission [10]. This identifies patients at risk for malnutrition or who are already malnourished and is well-recognized, but unfortunately, not performed everywhere. This secondary analysis aimed to investigate whether a simple method could be used to accurately screen for malnutrition in the hospital in a low-resource setting where there are few to no nutrition professionals. This approach included the use of three factors to classify risk of malnutrition: disease category, reported dietary intake and MUAC measurement. Non-mandatory nutrition screening upon hospital admission in Malawi is partly due to the lack of adequate nutrition professionals such as Registered Dietitians, to meet population needs. This study therefore sought to provide feasible solutions for low-resource settings such as Malawi in order to assist non-nutrition health professionals in the early referral of malnutrition at-risk patients to the current few dietitians. 

More than half of the patients in this study had reported poor dietary intake. In a study conducted by Peterson et al., the authors also found that the average daily energy and protein intake failed to exceed 50% of daily requirements for the entire population of critically ill patients [39,40]. In this study, those with organ-related disease and infectious disease had the highest rate of reported reduced dietary intake. In other studies that investigated dietary intake in hospitalized patients with similar disease categories, the proportion of patients with inadequate or reduced dietary intake was consistent with our findings [39,40,41,42,43,44,45]. In a prospective observational study conducted by Pekmezci et al., the researchers found that hospitalized patients with infectious disease had inadequate and reduced energy and protein intake. The authors concluded that routine dietetic assessment should be performed for all infectious disease patients, so that those at risk for malnutrition are identified early and nutritional intervention is started [44]. Additionally, though there are fewer data for the adult population, in a cross-sectional study conducted by Avilés et al., adolescent individuals at risk for cardiovascular disease had reduced energy intake, as well as limited intake of whole grains, vegetables, and fruit [46].

Of individuals who reported reduced dietary intake, the disease category of Burns + Surgical had the highest total percentage (84.6%) of moderate to severe malnutrition classifications, followed by infectious disease, organ-related disease and cancer. Additionally, individuals in the Burns + Surgical, Infectious Disease, and Organ-Related Disease categories, who reported borderline, decreasing dietary intake, had lower MUAC scores compared to their original mean MUAC scores. These results warrant further investigation to clarify if individuals in the disease categories of Burns + Surgical, Infectious Disease, and Organ-Related Disease with reported reduced dietary intake are at a higher nutritional risk compared to individuals who are just experiencing reduced dietary intake. At QECH, patients in the disease categories of Cancer, Infectious Disease, and Burns + Surgical, make up the small percentage of patients who receive either high or extra high protein diets. This could potentially explain why high rates of malnutrition were not observed in these disease categories for reported reduced dietary intake. In addition, we were originally surprised by the high rate of reported reduced dietary intake that we observed for participants in the Organ-Related Disease category. Perhaps edema led to early satiety, however, most of the patients with Organ-Related Diseases were on the regular diet with no extra protein and energy which contributed to the high rate of poor intake. These observations and our reasoning are only applicable for 65% of our study participants, since 35% of participants were from KCH.

The variables used in this study, dietary intake and MUAC, are both commonly used in hospital screening, hence the justification for their use [10,47]. Of the individuals who reported reduced dietary intake, 69.8% had moderate or severe malnutrition. Furthermore, of the individuals who reported no reduction in dietary intake, 48.7% were adequately nourished with no signs of malnutrition. There was no evidence in this dataset that suggested a relationship between dietary intake and MUAC measurements. However, there was a positive trend for reported poor dietary intake in those with a severely malnourished MUAC score. These findings suggest that this sample size (*n* = 91) was too small to show a significant relationship between dietary intake and MUAC. Therefore, the results above propose that with a larger sample size, there is the potential to establish statistical significance. It is also important to note that individuals with a borderline, decreasing dietary intake, had the highest rates of malnutrition (54.4%), compared to the other categories of no change in dietary intake or unable to eat. This further suggests that individuals who are beginning to experience a decrease in appetite might be at a higher risk for potential malnutrition.

The feeding of patients within hospitals in Malawi largely depends on foods provided from catering services, in part dependent on donations to supplement inadequate hospital meals, combined with food prepared and served by a patient’s designated guardian. Guardians are typically allowed to visit a hospitalized patient three times per day [48]. Assuming the patient consumes all foods served, all of the diets in this study exceeded 80% of the daily energy intake recommendations for a healthy individual, but with a gap of 100–250 kcals/day. In terms of protein, however, none of the diets met recommendations, especially considering the low biological value of foods served combined with increased nitrogen losses associated with injuries and infections. This study observed only critically ill patients, so it is important to keep in mind that all participants would most likely have some sort of increased energy and protein needs than our estimate. Although the estimated energy needs of the diets were fairly closely met, the quality of the food provided in all of the diets was not adequate for preventing and treating hospital malnutrition, nor optimal recovery, considering only three of the six food groups [protein, dairy, and grains] were served. 

Diseases and injuries in patients in the critical care setting, such as burns and trauma, are associated with increased protein metabolism, resulting in severe loss of muscle mass and fat stores, and overall increased nutrient requirements [11,49]. No patients, regardless of disease category, were prescribed a personalized diet that would best fit recovery for them. This finding is consistent with a study conducted in a Malawi Burn unit; Grundziak et al., reported that all patients received the same foods daily, no matter the age or severity of burn [50]. The shortfalls observed for the quality of food provided by the hospital could be attributed to the lack of standard guidelines for menu standardization or nutritional content of food [51,52,53]. This demonstrates the opportunity for improving hospital food service, types and amount of food; the need for trained nutrition professionals, and most importantly, the political will to modify and improve hospital policy that will allow for these changes.

### Limitations, Strengths, and Future Outlooks

This study is one of the first to look at nutrition Screening, delivery, and reported dietary intake of hospitalized Patients in Malawi. It was conducted by well-trained nutrition staff, including Registered Dietitians. There are some limitations, however, of note. The sample size used in this secondary analysis had inadequate power to detect differences due to missing data. Based on the observed variables in the MUAC data, in order to have a strong probability of significant effects, a sample size of 400 was needed to detect dietary effects and a sample size of 2000 was needed to detect disease-related effects. In addition, of the 100 adult participants, seven in the burn unit and one with infectious disease were unable to have their MUAC measurement taken due to arm burns or wounds, therefore, for MUAC, the sample size was further reduced. Due to the observational nature of the study, we were unable to control for different confounding factors such as socio-economic status, food sharing between patient and guardians and external food provision from guardians. Foods served were measured, however actual consumption was not, so the estimated energy and protein content served may not be what was consumed.

Due to the cross-sectional design of the study, MUAC and its associated exposures, reported dietary intake and disease state, were simultaneously assessed. The statistical model, however, looked at the effect of each variable after accounting for other variables, which would allow us to see an effect of dietary intake or disease state if it existed. Additionally, the aim was to look at MUAC and whether it was associated with poor intake or disease at the time the MUAC was measured. This is useful if the purpose is to establish whether low food intake (a relatively simple, easy, and less expressive measure) is a surrogate marker for another (more complex) measure like MUAC, which in turn is predictive of a poor hospital outcome.

For consistency, MUAC measurements for all participants were made on the left side. For a small number of participants this may have been their dominant arm which could have made a participant appear to be better nourished than they actually were, leading to an underestimation of malnutrition.

Ideally in the future, dietary intake should be collected as routine data and monitored closely in all critical care in-patients in tertiary hospitals in Malawi, treating all low-intake patients with a high index of suspicion for malnutrition. Particular attention should be paid to intake in patients with burns and surgical conditions as they were more prone to mild-severe malnutrition, based on our findings. Lastly, a multi-discipline approach in foodservice improvement is required; including in-service training and stakeholder engagement (Ministry of health) to support the budget of a balanced hospital diet to meet protein and micronutrient requirements. This, however, could begin with awareness through the collection of baseline data and publication of findings such as ours.

## 5. Conclusions

While hospital foods in Malawian public service central hospitals currently provide close to the estimated energy needs, high quality protein and diet diversity including fruit and vegetables are lacking. More than half of the patients in critical care units had reported poor dietary intake and there were significant rates of malnutrition among all four disease groups. Of those who reported reduced dietary intake, more than half had moderate to severe malnutrition, indicating that screening for malnutrition by asking about intake may be helpful for identifying those in need of nutrition intervention to reverse adverse outcomes of malnutrition. This is especially important for use in low-resource settings, such as Malawi, where there is a high clinical dietitian to patient ratio, as a way of triaging priority patients for nutrition care.

## Figures and Tables

**Table 1:** The demographics and anthropometrics for all adult patients.

Baseline Characteristics	Total (*n* = 100)	KCH (*n* = 35)	QECH (*n* = 65)
**Age (years)**	40.59 ± 14.64	40.50 ± 15.70	40.60 ± 14.14
19–34 n (%)	36	12 (34.3)	24 (36.9)
35–50 n (%)	46	17 (48.6)	29 (44.6)
≥51 n (%)	18	6 (17.1)	12 (18.5)
**Sex**			
Male, %	59.6	64.7	56.9
Female, %	40.4	35.3	43.1
MUAC, (cm) *	23.89 ± 4.16	24.75 ± 3.97	23.43 ± 4.22
Adequately nourished, %	38	40.6	36.7
Moderate malnutrition, %	20.7	21.9	20
Severely malnourished, %	41.3	37.5	43.3
**Weight, kg**	54.1 ± 10.8	56.8 ± 10.2	51.6 ± 10.9
**Height, cm**	163.4 ± 8.9	165.6 ± 8.5	-
**BMI (kg/m^2^)**	20.74 ± 4.33	21.99 ± 4.20	-

Note: Values are mean *±* standard deviation or total percentage; KCH–Kamuzu Central Hospital; QECH–Queen Elizabeth Central Hospital; * MUAC score missing for 7 burn patients and 1 infectious disease patient. Adequately nourished ≥25.5 cm; Moderate malnutrition = 23–25.4 cm; Severely malnourished <23 cm. Measurements for patients’ height were not available at QECH and therefore, BMI was not calculated.

**Table 2:** Disease categories by medical diagnoses.

Disease Category:	Burns + Surgical (*n* = 32)	Cancer (*n* = 27)	Infectious Disease (*n* = 20)	Organ-Related Disease (*n* = 21)
**Hospital:**	QECH (*n* = 16)	QECH (*n* = 27)	QECH (*n* = 14)	QECH: (*n* = 8)
	KCH (*n* = 16)	KCH (*n* = 0)	KCH (*n* = 6)	KCH (*n* = 13)
**Medical** **Diagnoses, *n***	Burn (16)	AML (1)	Acute gastroenteritis (2)	Abdominal pain (1)
	31%BSA (1)	Breast cancer (3)	HIV (3)	CKD (6)
	Crushed right hand (1)	Cervical cancer (4)	Meningitis (2)	Lesion (1)
	Crushed fibula (1)	Colon cancer (1)	Ovarian abscess (2)	Cerebral aneurysm (1)
	Leg fracture (1)	Esophageal carcinoma (4)	TB (3)	DKA (1)
	Debridement infection (2)	Hodgkin’s lymphoma (2)	TB meningitis (3)	RTA (2)
	Fournier’s Gangrene (1)	Non-Hodgkin’s lymphoma (2)	TB pericarditis (1)	HTN (3)
	Gastric perforation (1)	Mucinous carcinoma (1)	TB spine (4)	Liver disease (1)
	Neck mass (1)	Liposarcoma (1)		PID (1)
	Pressure sores (2)	Lung cancer (1)		Severe osteoarthritis (1)
	Traffic accident injuries (1)	Oral Kaposi Sarcoma (2)		Tetralogy of Fallot (1)
	Severe neck injury (2)	Parotid carcinoma (1)		Other (2)
	Stab wound (1)	Squamous cell carcinoma (2)		
	Brachial plexus injury (1)	Vulvar cancer (2)		
**Sex, *n***				
Male	26	7	12	14
Female	5	20	8	7
**Age, M *±* SD**	43.60 *±* 18.18	39.50 *±* 11.08	37.85 *±* 10.12	39.90 *±* 16.34

Note: Total body surface area (BSA); Acute myeloid leukemia (AML); Tuberculosis (TB); Chronic Kidney Disease (CKD); Diabetic ketoacidosis (DKA); Hypertension (HTN); Pelvic inflammatory disease (PID); Renal Tubular Acidosis (RTA). KCH–Kamuzu Central Hospital; QECH–Queen Elizabeth Central Hospital. Values are sample size, mean ± standard deviation or total percentage. M = mean; SD = standard deviation. Sex is missing for one patient in the Burns + Surgical disease category.

**Table 3:** Least-squares predicted mean MUAC.

	Mean MUAC (cm)	Standard Error
**Hospital**		
KCH	25.15	0.93
QECH	23.48	0.77
Sex *		
Female	24.68	0.87
Male	23.95	0.80
**Reported Dietary Intake**		
DI1 (no change)	25.00	0.82
DI2 (borderline)	23.60	0.68
DI3 (decreasing)	24.35	1.40
**Disease Categories**		
Burns + Surgical	23.81	1.25
Cancer	24.80	1.25
Infectious Disease	23.93	1.26
Organ-related Disease	24.74	1.11

Note: * Sex missing for one patient.

**Table 4:** Linear model output for MUAC (cm).

Source	DF	Sum of Squares	F-Ratio	Prob > F
**Model**	14	142.3	0.538	0.90
Hospital	1	34.4	1.821	0.18
Sex	1	7.2	0.379	0.54
Age	1	0.6	0.031	0.86
Disease Categories (DC)	3	9.9	0.176	0.91
Reported Dietary Intake (RDI)	2	34.9	0.924	0.40
DC × RDI Interaction	6	57.0	0.502	0.80
**Error**	76	1436.0	-	-
**Total**	90	1578.3	-	-

Note: Data for MUAC has a sample size of 91; data for all other explanatory variables have a sample size of 100.

**Table 5:** Disease category by reported dietary intake.

Disease Category	Burns + Surgical, *n* (%)	Cancer, *n* (%)	Infectious Disease, *n* (%)	Organ-Related Disease, *n* (%)	Prob ≤ P
**Reported dietary** **intake (DI)**					0.80
DI 1 (no change)	15 (46.88)	13 (48.15)	9 (45.00)	6 (28.57)	
DI 2 (borderline)	15 (46.88)	11 (40.74)	9 (45.00)	12 (57.14)	
DI 3 (decreasing)	2 (6.25)	3 (11.11)	2 (10.00)	3 (14.29)	

Note: Values are sample size and column percentages. *p*-value calculated using Fisher Exact test.

**Table 6:** Logistic model output.

Source	DF	Chi-Square	Prob > ChiSquare
**Model**	28	24.28	0.67
Hospital	2	1.616	0.45
Sex	2	3.57	0.17
Age	2	0.29	0.87
Disease Categories	4	8.18	0.09
Reported Dietary Intake	6	0.71	0.99
Disease Categories and Reported Dietary Intake Interaction	12	11.99	0.45

Note: Data for MUAC has a sample size of 91; data for all other explanatory variables have a sample size of 100.

**Table 7:** Nutritional composition of food provided by QECH.

	Porridge	Porridge (Milk)	Porridge (Milk and Egg)	Nsima	Cabbage (with Oil)
Serving Size	250 mL	250 mL	250 mL	530 gm	150 gm
Kcal	80	174	249	720	214.5
Protein (g)	3	7	14	21.6	3
Fat (g)	1	10.3	10.3	11.7	20
Carbohydrate (g)	15	23	23	135	7

**Table 8:** Nutritional composition of the different diets at QECH.

	Normal Diet	High Protein Diet	Extra High Protein Diet
Total Kcal	1949	2116.1	2117.5
Total Protein (g)	52.2	56.2	63.2
Energy from Protein (%)	208.8 (10.8%)	224.8 (10.7%)	252.8 (11.8%)
Total Fat (g)	64.4	73.7	73.7
Energy from Fat (%)	579.6 (27.8%)	663.3 (31.3%)	663.3 (30.9%)
Total CHO (g)	299	307	307
Energy from CHO (%)	1196 (61.4%)	1228 (58%)	1228 (57.3%)

Note: Measurements for these diets were taken on a non-meat day and represent an average over 4 days.

## Data Availability

All relevant data that support the findings of this study are within the paper.
